# Genomic variation predicts adaptive evolutionary responses better than population bottleneck history

**DOI:** 10.1371/journal.pgen.1008205

**Published:** 2019-06-12

**Authors:** Michael Ørsted, Ary Anthony Hoffmann, Elsa Sverrisdóttir, Kåre Lehmann Nielsen, Torsten Nygaard Kristensen

**Affiliations:** 1 Department of Chemistry and Bioscience, Aalborg University, Fredrik Bajers Vej, Aalborg E, Denmark; 2 Bio21 Molecular Science and Biotechnology Institute, School of BioSciences, The University of Melbourne, Parkville, Victoria, Australia; 3 Department of Bioscience, Aarhus University, Ny Munkegade, Aarhus C, Denmark; University of Georgia, UNITED STATES

## Abstract

The relationship between population size, inbreeding, loss of genetic variation and evolutionary potential of fitness traits is still unresolved, and large-scale empirical studies testing theoretical expectations are surprisingly scarce. Here we present a highly replicated experimental evolution setup with 120 lines of *Drosophila melanogaster* having experienced inbreeding caused by low population size for a variable number of generations. Genetic variation in inbred lines and in outbred control lines was assessed by genotyping-by-sequencing (GBS) of pooled samples consisting of 15 males per line. All lines were reared on a novel stressful medium for 10 generations during which body mass, productivity, and extinctions were scored in each generation. In addition, we investigated egg-to-adult viability in the benign and the stressful environments before and after rearing at the stressful conditions for 10 generations. We found strong positive correlations between levels of genetic variation and evolutionary response in all investigated traits, and showed that genomic variation was more informative in predicting evolutionary responses than population history reflected by expected inbreeding levels. We also found that lines with lower genetic diversity were at greater risk of extinction. For viability, the results suggested a trade-off in the costs of adapting to the stressful environments when tested in a benign environment. This work presents convincing support for long-standing evolutionary theory, and it provides novel insights into the association between genetic variation and evolutionary capacity in a gradient of diversity rather than dichotomous inbred/outbred groups.

## Author summary

Genetic variation is a prerequisite for evolution to occur. Quantifying, utilizing and understanding the role of this variation for the survival and persistence of populations is central to several research disciplines including evolutionary biology, animal and plant breeding and conservation genetics. Environments are changing at a rate unpreceded for millennia and rates of local and global extinction of populations and species are alarming. The ability to cope with environmental change through genetic changes is therefore key in order to adapt and survive. Here we provide insights into the association between genetic variation and evolutionary and demographic response to environmental stress. We do this based on results from a long-term experimental evolution study on 130 genome-wide sequenced inbred and outbred lines of vinegar flies (*Drosophila melanogaster*). We found 1) strong positive correlations between levels of genetic variation and evolutionary response, 2) that genomic variation is predicting evolutionary responses better than expected inbreeding levels, and 3) that lines with lower genetic diversity are at greater risk of extinction. This work presents convincing support for long-standing evolutionary theory, reinforces the importance on maintaining genetic variation in wild and domestic populations and pinpoints applied benefits of high-throughput sequencing.

## Introduction

Evolutionary theory predicts reduced capacity for adaptation in populations with small effective population size (N_e_) [1–4]. Causes include loss of genetic variation due to genetic drift limiting the potential for genetic adaptation [5–12] as well as an increased risk of deleterious alleles being linked to alleles under selection due to increased linkage disequilibrium in small populations [13–15]. Thus, many species and populations of conservation concern might simply be unable to adapt at a rate that is adequate to ensure their long-term survival and thus face extinction.

Theory predicts a positive and linear relationship between N_e_ and measures of genetic variation such as additive genetic variance (V_A_) in a population [8,15]. However, V_A_ and other variance components are typically difficult to estimate accurately using quantitative genetic approaches particularly in threatened species where large sample sizes and accurate pedigree information is rarely available [16]. For this reason, molecular tools are increasingly used in conservation genetics to assess genetic variance and population structure of threatened populations [17], commonly using putative neutral markers such as microsatellites, or large numbers of single nucleotide polymorphisms (SNPs). When available, historical and current population sizes are also used as a proxy of adaptive capacity. This is supported by studies on experimental populations showing that larger populations respond more to selection [18,19] and that large populations have a lower extinction risk compared to small populations [20,21].

A meta-analysis of studies from natural populations [22] suggested that there is a poor association between population size and adaptive potential, and numerous experimental studies concluded that V_A_ is not reduced with increasing inbreeding to the extent predicted from theory (see review [23]). However, the majority of these studies [22,23] investigated morphological traits, which tend to have high heritability estimates and uncertain connections to fitness. Thus, such studies might not correctly reflect genetic variation important for fitness in natural populations, where low heritabilities are common [24,25], and they also do not consider the evolutionary response of populations facing environmental challenges such as climate change, which are critical in assessing the resilience of threatened species. If adaptive capacity and resilience to extinction is to be evaluated for practical purposes, a shorthand way of measuring them is needed that is applicable to threatened populations [26]. However, the validity of proxies such as genome-level variability has surprisingly rarely been tested in a rigorous manner.

We set up a large-scale empirical study to investigate the effects of varying and ecologically relevant levels of inbreeding on the adaptive potential of *Drosophila melanogaster*. Specifically, we set up ca. 40 lines of each of three different levels of expected inbreeding as well as 10 outbred control lines and measured their baseline response to stressful medium reduced in nutrition and increased in acidity (**S1 Fig**). We obtained molecular estimates of genomic variation (nucleotide diversity, π) of pooled samples consisting of 15 males of each line in the generation before we started the experiment using genotyping-by-sequencing (GBS) technology. Each line was then reared on this line-specific stressful medium for 10 successive generations, during which we measured productivity, body size, and extinction in each generation. These data generated novel insights into the association between inbreeding and the rate of evolutionary adaptation in stressful environments. After 10 generations of experimental evolution on the stressful medium, we quantified egg-to-adult viability on both the stressful medium and a benign medium to assess evolutionary responses and to identify potential trade-offs in adaptation. These data enabled quantification of the relationship between evolutionary responses and genomic variation in the individual lines.

## Results

### Extinction risk was associated with low genomic variation

**Fig 1:**
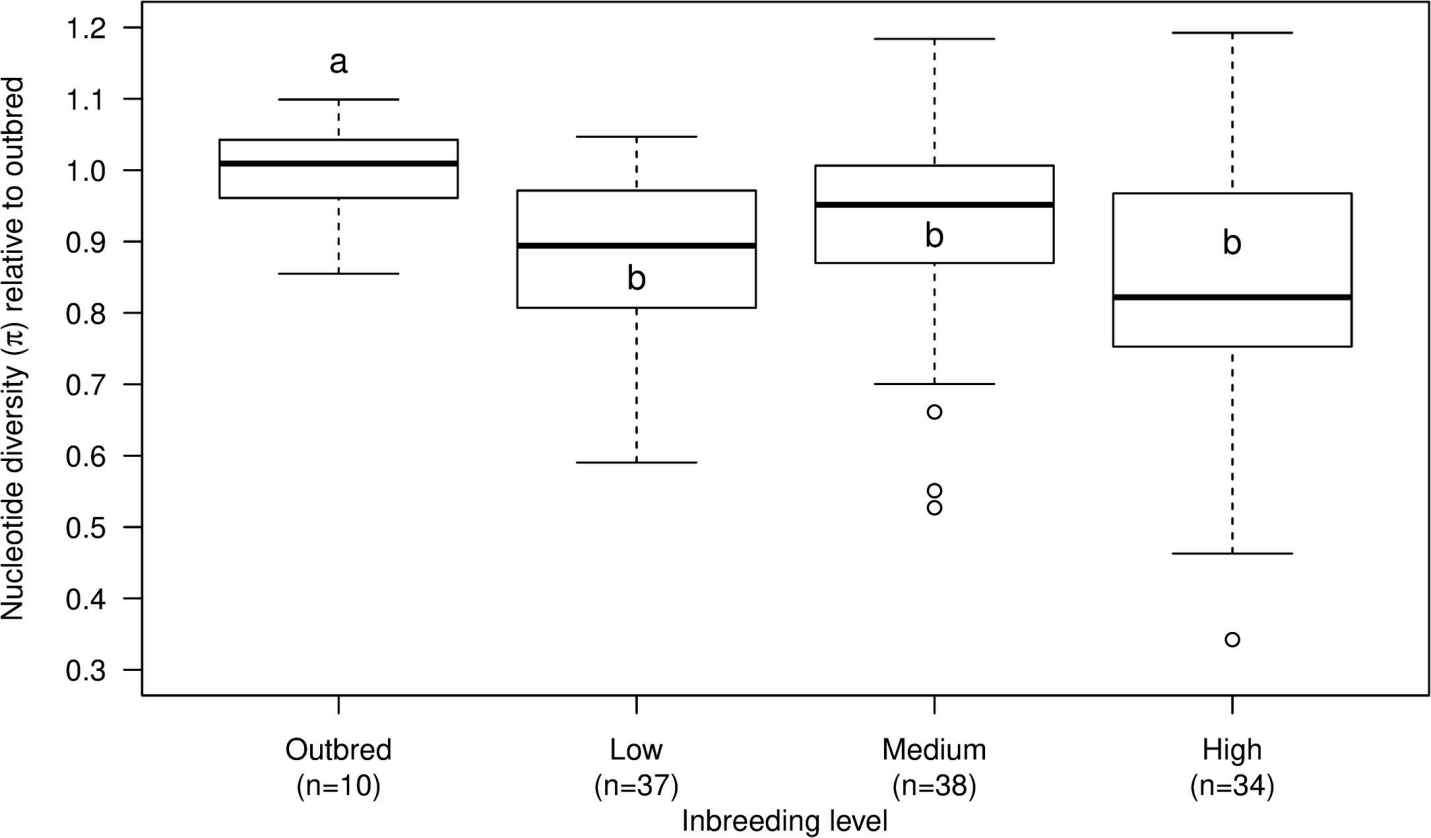
Nucleotide diversity was lower in inbred lines of all inbreeding groups than in outbred lines. Boxplot of nucleotide diversity (π) of the experimental lines of the three expected inbreeding levels (Low, Medium, and High) and the outbred lines expressed here as relative to the mean of the outbred lines. Expected inbreeding coefficients (F) of the Low, Medium, and High inbreeding levels are 0.125, 0.219, and 0.381, respectively. Letters denote significant differences in means as determined by Welch’s t-test, i.e. the mean π of outbred lines was significantly higher than the mean π of inbred lines in all three inbreeding groups. Numbers in parenthesis (n) show the number of lines within each inbreeding level for which π could be obtained (total n = 119). SE is: 1.621, 1.363, 1.672, and 2.383 for OB, Low, Medium, and High, respectively.

**Fig 2:**
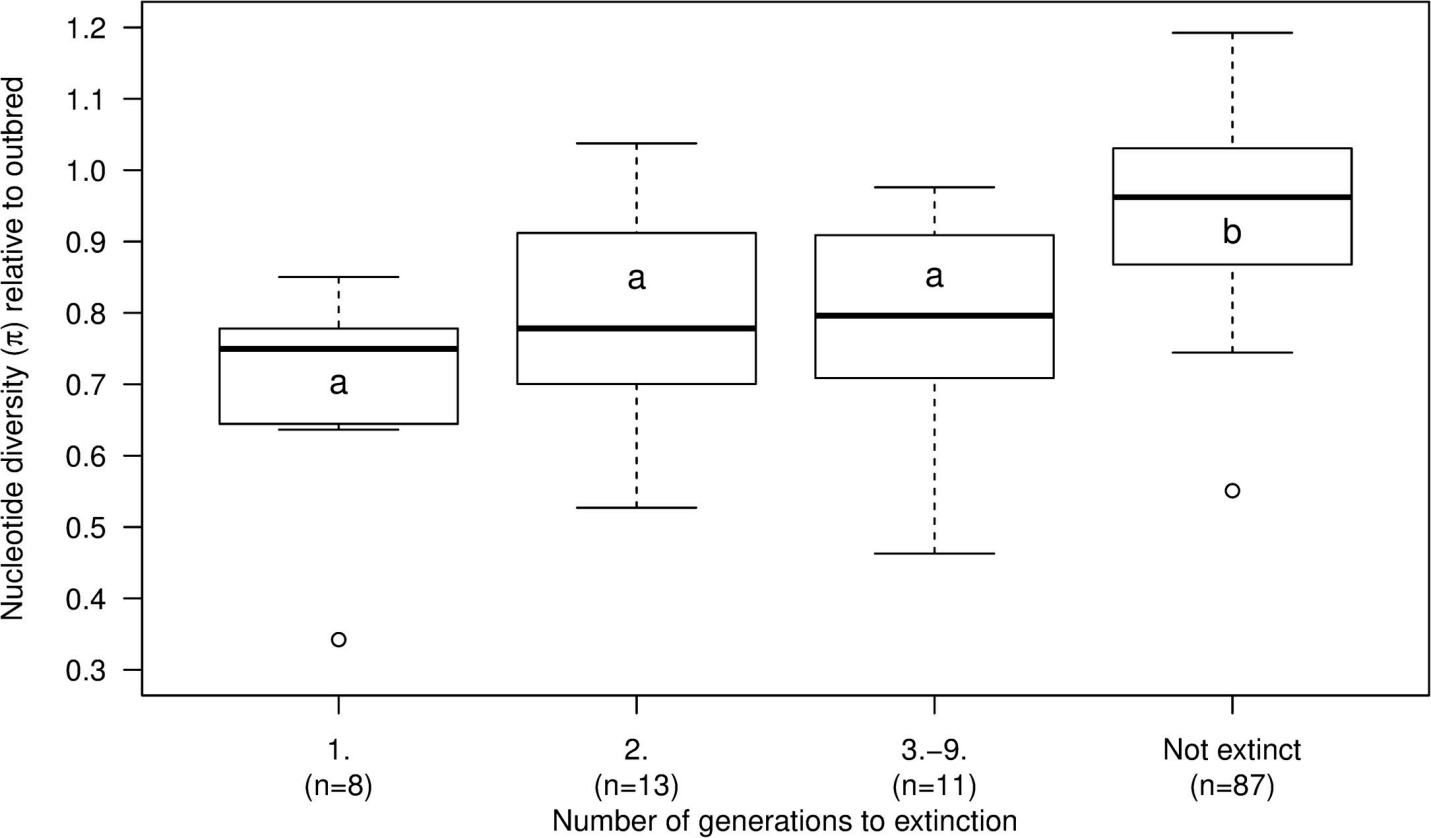
Low genomic variation was associated with elevated extinction risk. Boxplot of nucleotide diversity (π) of the experimental lines that went extinct in either the first generation, the second generation, or in the third to the ninth generation, as well as for lines that did not go extinct, with π expressed relative to the mean π of the outbred lines. Numbers in parenthesis (n) show the number of lines in each group for which π could be obtained (total n = 119). Letters denote significant differences in mean π as determined by Welch’s t-test, i.e. the mean π of lines that went extinct during the experiment (generations 1–9) was significantly lower than the mean π of lines that did not go extinct.

We started with 122 inbred lines and 10 control outbred lines of *Drosophila melanogaster* (**S1 Table**). Inbred lines from all population bottleneck levels had significantly lower nucleotide diversity than the outbred lines (**Fig 1**; *t*(39)<3.91; P<0.05). While all outbred lines persisted, 37 of the inbred lines went extinct (see Materials and Methods section for our definition of extinction) during the experimental evolution procedure. The extinction risk of the populations was related to nucleotide diversity; the nucleotide diversity of lines that went extinct were lower while those that persisted for all 10 generations of the experiment had higher nucleotide variation (**Fig 2**; *t*(14)<-3.23; P<0.01).

### Nucleotide diversity was a better predictor of evolutionary responses than population bottleneck history

**Fig 3:**
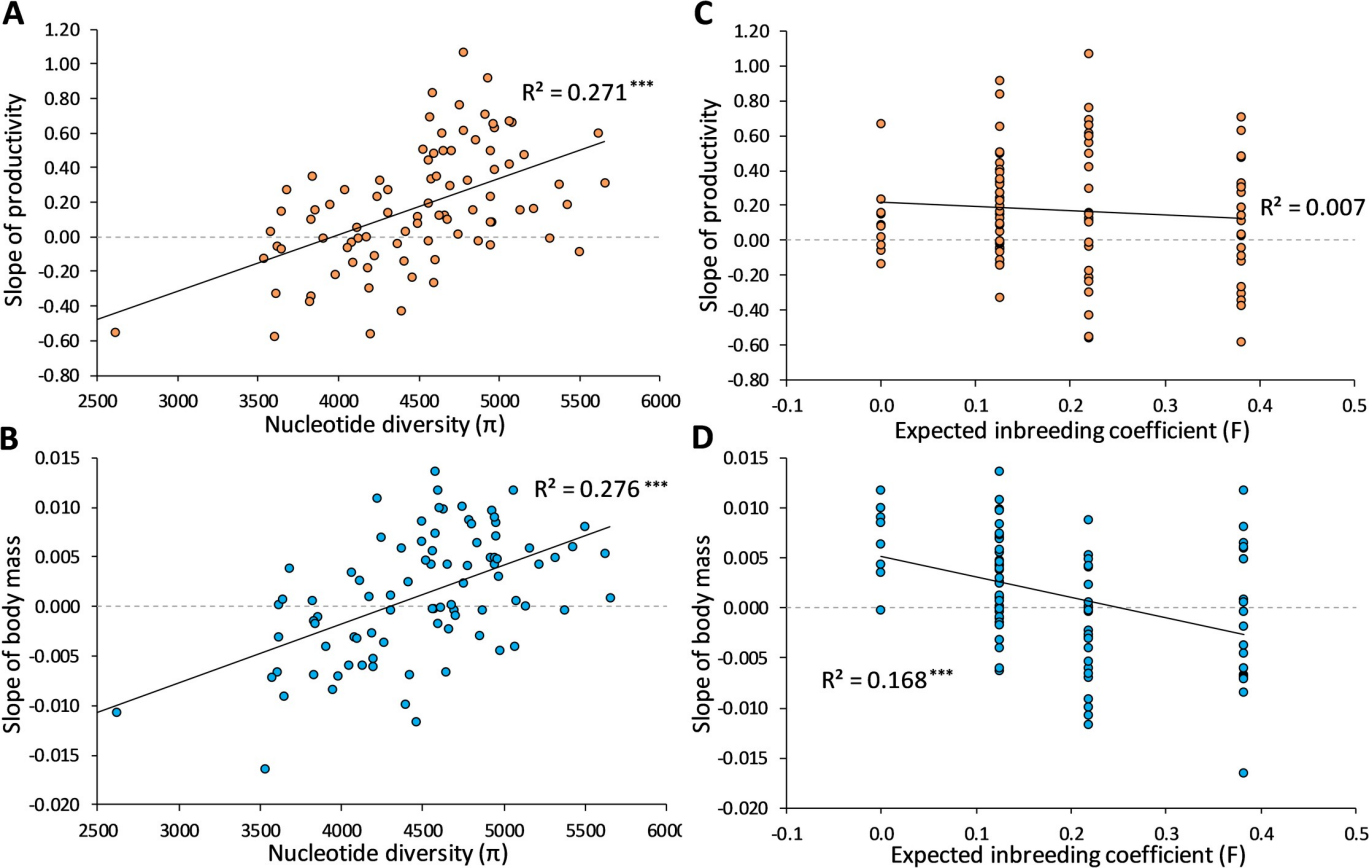
Genomic variation predicts evolutionary responses in productivity and body mass better than expected inbreeding coefficients. Correlations between nucleotide diversity (π) (**A-B**) or expected inbreeding coefficient (F) (**C-D**) and slope of evolutionary responses for productivity (Slope of productivity; **A and C**) and body mass (Slope of body mass; **B and D**). Black solid lines represent linear regressions to visualize the relationships, and R^2^ values are shown followed by asterisks denoting significant correlations; * P<0.05, ** P<0.01, *** P<0.001. We only considered slope for lines that did not go extinct, to ensure that unreliable slope estimates generated from information across e.g. just 2 generations were not biasing the results. Further, only lines with available nucleotide diversity measures were included. In total this yielded 87 lines for computing the correlations. Dashed grey lines are shown at slope = 0 for comparison.

Evolutionary response was measured by changes in three traits: productivity, dry body mass and egg-to-adult viability (**S2–S4** **Figs** and **S2–S4 Tables**). For productivity and dry body mass, we quantified the evolutionary response of each line as the slope of a linear regression of line means across generations. Evolutionary responses (slopes) in both traits showed an association with nucleotide diversity; we found a positive association between π and the slope of both productivity (**Fig 3A**; *F*_1,85_ = 31.56; P<0.001) and body mass (**Fig 3B**; *F*_1,85_ = 32.34; P<0.001), with R^2^ values of 0.27 and 0.28 for productivity and body mass, respectively. These associations were similarly positive within the predefined inbreeding groups (**S5 Fig**).

The expected inbreeding levels estimated based on the history of population bottlenecks were also associated with the magnitude of the evolutionary responses observed for some traits (**S3 and S4 Figs**). However, expected inbreeding coefficients (F) were less accurate in predicting adaptive evolutionary responses than genomic variation. There was no association between expected F and the evolutionary response in productivity (**Fig 3C**; *F*_1,85_ = 0.227; P = 0.64), but expected F was associated with the evolutionary response in body mass (**Fig 3D**; *F*_1,85_ = 16.22; P<0.001). Overall, expected F had lower explanatory value than nucleotide diversity, as reflected in lower R^2^ values of 0.01 and 0.17 for productivity and body mass, respectively. This was also tested with an encompassing model test, where the two competing models (π or F as the predictor variable) that are used to predict the evolutionary response, are nested in a combined model. This test similarly showed that π explained a greater proportion of the variation in the combined model than expected F for body mass (*F*_-1,84_ = 23.81; P<0.001 for expected F, and *F*_-1,84_ = 40.99; P<0.001 for π). For productivity using F as a regressor added no explanatory value to the combined model (*F*_-1,84_ = 0.30; P = 0.58). These patterns are probably due to the variation in nucleotide diversity generated by the different population treatments (**Fig 1** and **S5 Table**). The selection responses of both productivity and body mass were independent on which acid level the lines were selected on (see methods; ANOVA; *F*_1,94_ = 0.72; P = 0.40 for productivity and *F*_1,21_ = 0.68; P = 0.42, for body mass).

### Evolutionary responses of viability suggest trade-offs

**Fig 4:**
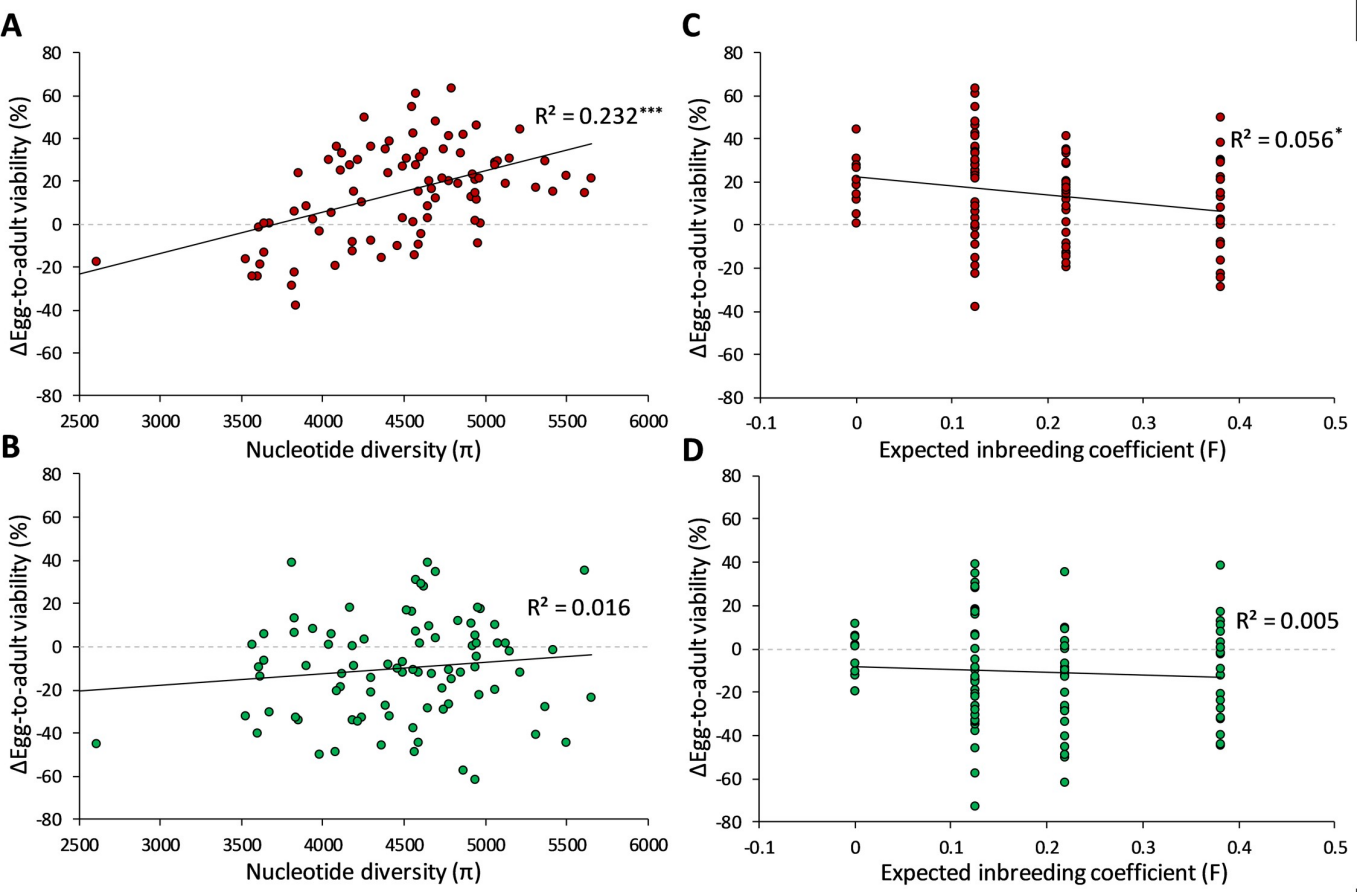
Genomic variation predicts evolutionary change in egg-to-adult viability in the stressful environment. Correlations between nucleotide diversity (π) (**A-B**) or expected inbreeding coefficient (F) (**C-D**) and changes in egg-to-adult viability (Δ). Δ was calculated as the difference in egg-to-adult viability measured at generation 10 (Gen 10) and generation 0 (Gen 0) on the stressful medium on which the specific line had been reared on throughout the experiment (**A and C**), and on the benign medium (**B and D**). Thus, a positive change represents lines where the viability is higher after compared to before the experiment at the respective mediums. Black solid lines represent linear regressions to visualize the relationships, and R^2^ values are shown followed by asterisks denoting significant correlations; * P<0.05, ** P<0.01, *** P<0.001. We only considered difference in viability for lines that did not go extinct, and lines with available nucleotide diversity measures; in total this yielded 87 lines for computing the correlations. Dashed grey lines are shown at slope = 0 for comparison.

Egg-to-adult viability was assessed before and after 10 generations of rearing on the stressful medium as well as on a benign medium (**S1 Fig**). Outbred lines and all inbred groups, except the highest inbred ones, exhibited a significantly increased mean viability on the stressful medium after generation 10 when compared to the start of the experiment (**S4 Fig**). Conversely, all inbred groups performed significantly worse in terms of viability on the benign medium, and only the viability of outbred lines were not significantly different on the benign medium in generation 10 when compared to viability before rearing at the stressful medium (paired two-sample t-test; *t*(9) = 1.13; P = 0.29). Similar to evolutionary responses in productivity and body mass, we observed a positive association between π and the difference in egg-to-adult viability between generation 0 and 10 when tested on the stressful medium (**Fig 4A;** *F*_1,85_ = 22.90; P<0.001). On the benign medium we found no association between π and the difference in egg-to-adult viability in generation 0 and 10 (**Fig 4B;** *F*_1,85_ = 1.43; P = 0.23). There was some association between the expected inbreeding coefficients (F) and the difference in egg-to-adult viability on the stressful medium (**Fig 4C;** *F*_1,85_ = 5.74; P = 0.018), but no association between F and the difference in egg-to-adult viability on the benign medium (**Fig 4D;** *F*_1,85_ = 0.93; P = 0.34). Similar to productivity and body mass, expected F overall had lower explanatory value than nucleotide diversity, as reflected in lower R^2^ values of 0.056 and 0.005 for the stress and benign medium, respectively, which was also confirmed by the encompassing model tests (e.g. on the stressful medium; *F*_-1,84_ = 7.31; P = 0.008 for expected F, and *F*_-1,84_ = 24.59; P<0.001 for π).

## Discussion

We performed a highly replicated experimental evolution experiment to investigate the relationship between population bottlenecks, loss of genetic variation, evolutionary potential and extinction risk across a gradient of genetic diversity. We found strong evidence that population bottlenecks resulted in loss of genetic variation, even for low levels of inbreeding (**Fig 1**), and that population bottlenecks and low genetic variation was associated with increased extinction risk (**Fig 2, S1 Table**). This is in agreement with numerous experimental studies showing that inbreeding and low effective populations sizes elevate the risk of extinction [20,27–31].

We further provide clear evidence that population bottlenecks and the resulting loss of genomic variation severely impedes evolutionary responses in key fitness traits. These relationships support classical evolutionary theory and emphasizes the importance of maintaining high N_e_ in natural populations. Our findings are in contrast with the apparently poor associations between population size and adaptive potential suggested by recent reviews [e.g. 21,22]. Although several studies have investigated the effects of population bottlenecks on the genetic and phenotypic variation of quantitative traits, only a handful of studies have assessed the consequences of population bottlenecks on evolutionary capacity [e.g. 18,32,33], and none of these have obtained molecular estimates of genetic variation or provided the level of replication presented here.

We found that expected inbreeding coefficients (F) overall had lower explanatory value than nucleotide diversity (π), suggesting that population bottleneck history is less useful in predicting adaptive evolutionary responses compared to genomic variation (**Fig 3; S2–S6 Figs**). Consequences of population bottlenecks are expected to be line specific and deviations from projected impacts on genetic variance components is often found in experimental studies [34–36]. This is not picked up when impacts of inbreeding on genetic variance is based on population history. However, our molecular data provide information on line-specific impacts of population bottlenecks. For instance, we detected only a slight decrease in mean diversity with increased expected levels of inbreeding, but we observed a marked decline in the minimum genetic diversity of lines, accompanied by no change in maximum diversity (**Fig 1** and **S5 Table**). This suggests that inbreeding does not necessarily result in reduced nucleotide diversity as some inbred lines are as genetically diverse as the outbred lines. Other studies have also shown that small bottlenecked populations can have higher than expected levels of genetic variation that can be selected on due to the influence of linkage disequilibrium and other gene interactions causing e.g. associate overdominance [37,38], and/or balancing selection favoring heterozygotes [36].

The poorer performance of expected F relative to nucleotide diversity in explaining adaptation can be due to several factors. Firstly, there is likely a high variance in the pedigree structure (which is not known) and thus in realized N_e_ among replicates for each bottleneck treatment. This can be due to differences between lines in the realized number of breeders and their mean and variance of reproductive success in each bottleneck generation [39]. Secondly, the role of chance in the sampling of alleles during the experimental bottlenecks, i.e. stochastic processes associated with small population size, could also be an important driver of our results [39,40]. Both of these factors will likely act in combination.

The importance of genetic drift and inbreeding, respectively, for explaining lower fitness and reduced response to selection in lines kept at low N_e_ cannot be distinguished with our design. However, our molecular data provide evidence that genetic drift, and not inbreeding has led to a reduction in π, as inbreeding does not change allele frequencies. This likely explains reduced slopes (responses to selection). However, inbreeding might expose recessive or partly recessive deleterious alleles, increasing the efficiency of selection against such alleles. This purging might be dependent on levels and rates of inbreeding [36,41] and the net impact on responses to selection is unclear. We also note that we cannot distinguish whether populations went extinct due to inbreeding depression or an inability to respond to the imposed selection. Inbreeding depression and failure to respond to selection likely acted synergistically in determining population viability.

The results for egg-to-adult viability suggest a trade-off between performance in the stressful and benign environments. Nucleotide diversity was positively associated with the observed evolutionary change in viability on the stressful medium, but not on the benign medium (**Fig 4**). This is in agreement with the commonplace evolutionary trade-off hypothesis that increased fitness in the environment of selection is accompanied by a decrease in fitness in other environments [42]. In fact, this trade-off was related to the level of inbreeding (**S4 Fig**). Thus, for the inbred lines, evolutionary adaptation to poor environmental conditions led to changes that were maladaptive under benign conditions, whereas evolutionary adaptation seemed to carry no cost for the outbred lines. This suggests that inbreeding is accompanied by the accumulation of deleterious alleles affecting survival in the benign medium. Interestingly, by relating evolutionary responses with a gradient of nucleotide diversity, we show that, regardless of expected inbreeding, high nucleotide diversity is associated with a stronger selection response under the stressful conditions, but decoupled with correlated responses in the benign environment, meaning that high diversity lines respond faster to selection but at similar cost.

We acknowledge that measures of genomic variation based on nucleotide diversity capture only one aspect of the evolutionary potential in populations; other factors like population specific levels of linkage disequilibrium are also critical [10,38] and may contribute to the variation not accounted for in our study. However, our results reemphasize the role and importance of N_e_ in determining evolutionary potential, and indicate that molecular tools provide robust estimates of genetic variation and evolutionary potential in natural populations. In summary, we show that genomic variation was more informative in predicting evolutionary responses than population history measured as expected inbreeding levels. Therefore, we advocate that molecular measures of inbreeding and genetic variation should be used when assessing natural or domestic populations of conservation concern. In conservation biology, genome-level variation as a proxy for adaptive capacity and extinction resistance has practical applicability for rapidly assessing the vulnerability of populations [26], such as under climate change [43]. By obtaining genome-wide estimates of genetic diversity, we were able to quantify the association between the genetic diversity and evolutionary response, resulting in patterns which would not have emerged using only estimates of inbreeding coefficients based on N_e_. Given the alarming increase in fragmented and isolated populations of small size and the rapidly changing environmental conditions, we reiterate that a focus on using molecular tools to assess genetic variation within and between populations and to assess connections among small and genetically depauperate populations should be a prime focus in applied evolutionary and conservation genetics.

## Materials and methods

### Fly stock and maintenance

The *D*. *melanogaster* population used in this study originated from flies caught at Oakridge winery in the Yarra Valley, Victoria, Australia (37°41'15"S 145°27'27"E) in April 2016. A total of 232 wild caught inseminated females each contributed with an equal number of offspring (five males and five females) to the establishment of a mass bred population. This population was maintained at a minimum size of 1000 individuals at 19°C in a 12:12 L:D photoperiod for 4 generations prior to establishing the lines used in the experiment. To control density in the generations prior to establishing the lines used in the current study, 200 parental flies laid eggs for 4–5 days in 175 mL bottles with 50 mL standard *Drosophila* sucrose-yeast-agar medium. Nipagen (10 mL/L) and acetic acid (1 mL/L) were added to the medium to control fungal growth. At the beginning of the inbreeding procedure the flies were moved to 25°C and a 12:12 L:D photoperiod and maintained under these temperature and light conditions for the remainder of the experiments.

### Inbreeding procedure

We created lines of each of three expected levels of inbreeding (denoted Low (L), Medium (M) and High (H)) by controlling the number of adult flies (N = 4) in successive generations of bottlenecks (**S1 Fig**). In all bottlenecks, we assume that the four flies contribute equally to the next generation. To set up inbred lines from the mass bred population, virgin flies were sorted less than 8 hours after emergence under light CO_2_ anaesthesia and for each line two males and two females were transferred to a 27 mL vial with 10 mL food. After three days, the flies were tipped to another vial and discarded after another three days. To generate lines with low, medium, and high levels of inbreeding, this procedure of sorting two male and two female virgin flies was followed for a total of 2, 3 and 5 succeeding generations, respectively (**S1 Fig**). The lines with different expected levels of inbreeding were set up asynchronously, so that they reached the desired inbreeding level at the same time, after which they were flushed to a population size of minimum 200 individuals that were maintained in bottles. We assume there was no inbreeding in the founding population and that N_e_ was equal to census size (N), thus, the estimated coefficient of inbreeding (F) at a given generation (t) with 2 breeding pairs, i.e. N_e_ = 4, was calculated according to [44]:

$$F_{t}=\frac{F_{t‐1}+\left(1‐2F_{t‐1}+F_{t‐2}\right)}{2N_{e}}$$

The estimated F of the low, medium and high inbreeding lines were 0.125, 0.219 and 0.381, respectively. The inbred lines went from mass bred (~1000 individuals) through generations of bottlenecks (2, 3 or 5) of 4 individuals to a population size flushed to 200 individuals. The N_e_ of the three inbred populations were estimated as the harmonic mean of the fluctuating population sizes over t generations [39]:

$$N_{e}=t∙{\left(\sum_{i=1}^{t}\frac{1}{N_{i}}\right)}^{-1}$$

The estimated N_e_ of the low, medium and highly inbred lines were 5.6, 6.6 and 7.9, respectively. It should be noted that these are not pedigree-based N_e_ estimates, because we do not have information on the realized number of breeders and their mean and variance of reproductive success in each generation. Some lines were lost due to extinction or the death of one or more of the four flies during breeding. Therefore, surplus lines were set up to make it plausibly that >40 lines per inbreeding level reached the expected level of inbreeding. The total number of lines after the inbreeding procedure was approximately 40 lines per inbreeding level plus 10 outbred lines (hereafter referred to as outbreds 1–10; OB1-OB10) totalling 132 lines at the beginning of the experiment (**S1 Table**).

### Baseline characterization of stress response

During the 10 generations of rearing at stressful conditions the lines were exposed to medium which was reduced in yeast concentration and acidified with acetic acid. Preliminary range finding tests of yeast and acid concentrations revealed large line-specific differences in responses to the treatments between and within inbreeding levels. Therefore, we investigated egg-to-adult viability in each of the 132 lines exposed to varying stress levels in order to start the experiment at a line specific stress level that yielded an approximately similar effect on viability. We did that by allowing approximately 20 flies (4–5 days old) from each line to lay eggs on plastic spoons filled with 1.5 mL standard medium and placed in a vial. After 12 h, 15 eggs were picked from the spoons and transferred to each of 5 vials with 10 mL of the respective medium per line, while carefully avoiding transferring egg-laying medium to the low nutrition vials. In total 49,500 eggs were distributed to 3,300 vials. We tested all lines including the outbred lines on four different stressful low-nutrition-low-pH medium consisting only of 9.5 g/L yeast, 16 g/L agar and 1.0, 2.5, 5.0 or 10.0 mL/L acetic acid, plus a benign control consisting of standard *Drosophila* sucrose-yeast-agar medium. The acetic acid concentration yielding the survival closest to 50% egg-to-adult viability was selected as the acid concentration used in the experimental evolution study to ensure, as closely as possible, that the initial strength of selection was consistent across lines. Otherwise it would not have been possible to compare the rate of evolution across the lines linked to adaptive variation (they would have reflected selection intensities). The results of the baseline characterization of egg-to-adult viability can be seen in **S4 Table**.

### Rearing for 10 generations on stressful medium

Based on the baseline characterization of egg-to-adult viability, lines were exposed to one of two different stress levels from the beginning of the experiment, and for each line this level of stress was maintained throughout the 10 generations. A total of 122 inbred lines (42 low, 40 medium, and 40 high; S1 Table) were started on stressful medium containing 9.5 g/L yeast, 16 g/L agar and either 1 mL/L (84 lines) or 2.5 mL/L (38 lines) acetic acid (**S4 Table**). The 10 outbred control lines were all exposed to the 2.5 mL/L acetic acid medium. At the beginning of the experiment approximately 200 adult flies (5 days old) per line were transferred to 175 mL bottles containing 50 mL of the respective stressful medium. Here they laid eggs for 48 h and were then tipped to a new identical bottle and laid eggs for another 48 h before being stored in absolute ethanol and counted. Flies that had died on the medium in either of the two bottles were also counted. Emerging adult flies were collected from first day of emergency and the following days and transferred to a 175 mL bottle with 50 mL standard medium sprinkled with dry yeast, to recover before again being exposed to the stressful medium. This was done to stimulate egg-production and to reduce maternal carry-over effects of the low-nutrition medium, i.e. a cumulative reduction of egg-production throughout the generations. When approximately 200 emerged flies from a line had been collected and all flies had experienced a minimum of 5 days recovery, they were transferred to a new bottle with stressful medium similar to the previous generation, and the egg-laying procedure was repeated. This was carried out over 10 consecutive generations.

### Phenotypes assessed

#### Productivity

All flies that emerged from the bottles were stored in ethanol and counted to provide an estimate of total number of flies produced by each line in each generation. This included the collected flies that contributed to the next generation after the egg-laying periods, and all surplus flies that emerged. All flies were considered emerged from a bottle when no flies had emerged from a given bottle for 10 consecutive days (because of the poor nutritional quality flies often emerged over a long period). We computed a total productivity measure (adult flies produced per female per day) to account for slight deviations in egg-laying time and in number of females [45]. We assumed a 1:1 sex ratio, thus the total number of egg-laying females establishing a new generation was half of the total number of flies (approximately 200 flies in total = 100 females per generation). If 200 individuals could not be collected a line was considered extinct.

#### Dry body mass

From each line and each generation, the dry body mass (hereafter referred to simply as body mass) of 15 males were measured by drying the flies at 60°C for 24 h (for exact numbers see **S3 Table**). To prevent re-absorption of humidity, the samples were transferred to a desiccator with silica gel after drying, and from there flies were transferred and measured individually on a Quintix35-1S laboratory scale with a resolution of 0.01 mg (Sartorius, Göttingen, Germany). In total 15,343 males were individually assessed for body mass.

#### Egg-to-adult viability assessed after 10 generations of rearing in the stressful environments

To assess the evolutionary response in the ability to survive from the egg to the adult stage we assessed egg-to-adult viability after 10 generations of rearing on the stressful medium in the lines that did not go extinct (n = 96). This followed the same procedure as for the assessment of egg-to-adult viability in the initial baseline characterization of the response to stress. Viability was determined both on the stressful medium to which the specific line had been exposed, and on a benign medium. The numbers of replicate vials were increased to 10 per line per environment. For this assessment of egg-to-adult viability 28,800 eggs were distributed to 1920 vials. For each line, the results were compared to the initial response as determined before starting the experiment.

### Assessment of genetic diversity by Genotyping-By-Sequencing (GBS)

#### DNA extraction

From the generation immediately prior to starting the 10 generations on stressful medium, a sample of 15 randomly collected males (~15 mg wet weight) from each line was homogenized in a tube with three sterile 2 mm glass beads by subjecting it to 2x6 s cycles at 6500 rpm using a Precellys mechanical homogeniser (Bertin Technologies, Montigny le Bretonneux, France). DNA was extracted with DNeasy Blood & Tissue Kit (QIAGEN, Hilden, Germany) following a specialized protocol for insect tissues according to manufacturer’s instructions. Concentration and purity of extracted DNA was assessed on a 1% agarose gel and on a NanoDrop 1000 spectrophotometer (Thermo Scientific, Waltham, MA, USA).

#### Preparation of GBS libraries

5’ and 3’ barcoding adapters were designed as previously described [46]. Adapters were designed to contain a 3 bp overhang complementary to the overhang generated by *Ape*KI (CWG). 5’ adapters also contained eight different internal 4 to 8 bp barcode sequences, as described in [47], while 3’ adapters contained 12 different 6 bp barcode sequences compatible with standard Illumina sequencing multiplexing, enabling a 96 multiplexing system. Adapters were designed so that the *Ape*KI recognition site did not occur in any adapter sequence and was not regenerated after ligation to genomic DNA.

DNA samples were digested with *Ape*KI (NEB) and ligated to adapters according to the 96 Plex GBS protocol developed by [47] with minor modifications. Sets of 66 differently barcoded samples were combined in two pools and purified using Agencourt AMPure XP PCR purification system (Beckman Coulter, Indianapolis, IN, USA). Restriction fragments from each library were amplified in 50 μL volumes containing 4 μL pooled DNA fragments using Phusion High-Fidelity PCR kit (Thermo Scientific). Primer design and temperature cycling was performed according to the protocol developed by [47]. Libraries were purified as before and diluted to 2 nM as determined by Qubit (Thermo Scientific, Waltham, MA, USA). Single-read sequencing (200 bp) was performed on a rapid run flow cell on a HiSeq 2500 (Illumina, San Diego, CA, USA).

#### Nucleotide diversity

Sequenced reads were demultiplexed using fastq-multx [48] sorting the data into separate files, removing the barcode, and discarding reads that did not perfectly match any of the barcodes. To ensure equal chance of detecting variants across all samples, 1,500,000 reads were sampled from each sample and mapped to the reference genome of *Drosophila* r6.14 using the CLC Genomics Workbench v9.5.2 (Qiagen, Hilden, https://www.qiagenbioinformatics.com) using default parameters, no masking of repetitive regions, a length fraction of 0.5 and a similarity fraction of 0.8. Non-specific matches were ignored and thus not included in the analysis. Following mapping, variants were called with the Basic Variant Detection module in the CLC Workbench using a min coverage of 10, max coverage of 600, minimum count of 1 and a min frequency of 10%. Again non-specific matches were ignored. The variant table for each sample was exported. Using a custom Bash script, the variants mapping to autosomes 2, 3 and 4 were used to calculate nucleotide diversity (*π*) for each variant locus: *π* = *p*∙(1−*p*). SNPs different from the reference but monomorphic across all samples were not included. Thus, we have defined *π* as similar to expected heterozygosity (*H*_*e*_) at the variable genomic positions throughout the present work. Our measure of genetic variation, was estimated for each sample by summing π over all variant loci. In total, π was estimated for 119 lines (**Fig 1, S6 Table**) (π could not be determined for all lines (see criteria above)).

Estimation of variation of π at specific sites across populations is subject to some controversy. Several studies employ pooled sequencing for acquiring population-level SNP frequency, and have found the method to perform as well as individual based sequencing [49,50] or perhaps even better [51]. Contrary to Anand et al. [50] who found that their SNP frequency estimations based on pooled sequencing were reliable, Lynch et al. (2014) [52] have argued that an inflated number of false positives in differentially identified polymorphic sites can result from frequency estimates of variation of rare variants based on low read coverage. In this study it is important to note, that while indeed sites at low read coverage (coverage range 10–515 across all sites) is used to estimate π, no comparison at individual SNP sites across populations have been performed. Instead the average nucleotide diversity of the sample is estimated from a large number of sites within a sample for each treatment (number of SNPs range 11267–35496, **S6 Table**). While the estimation at each site may be imprecise due to randomness of sampling of rare variants as argued by Lynch et al. (2014) [52], the sampling bias should be the same in all bottleneck treatments, when the read coverage distributions are similar. To verify this, a Kolmogorov-Smirnov test on the cumulative distributions of read coverage for each bottleneck treatment was performed (**S7 Fig**). At α = 0.05, the distributions were not significantly different between population bottleneck treatments, justifying using mean π, as a measure of overall nucleotide diversity.

### Statistical analysis of phenotypes

All statistical analyses were performed in R v. 3.4.0 [53]. Body mass data was analysed with a linear model. Productivity data were analysed with a generalised linear effect model (GLM) with a Poisson distribution. Egg-to-adult viability data were analysed with a GLM with a binomial response and a logit link function. We detected overdispersion in the viability GLM and corrected for this using a quasi-binomial linear model. In all linear models the trait (body mass, productivity or viability) was included as the response variable and either generation (in cross-generation comparisons) or bottleneck treatment (i.e. expected F in within-generation comparisons) as the predictor variable. In the analysis of the difference in viability from before (F_0_) and after the experiment (F_10_), the SE of the difference was calculated from the variance sum law [54]: $\sqrt{\frac{\sigma_{F10}^{2}}{n_{F10}}+\frac{\sigma_{F0}^{2}}{n_{F0}}}$, where σ^2^ are the variances and n are the sample sizes of viability measures from F_10_ and F_0_. We calculated between line coefficients of variation (CV = SD/mean) for each generation for each inbreeding level as a measure of divergence within and between levels of inbreeding. In analyses of productivity and egg-to-adult viability across generations general linear mixed models (GLMMs) were employed using the R-package ‘lme4’ [55] with line included as a random effect, as measures of a given line across generations are not independent. Body mass was analysed across generations with repeated measures (RM) ANOVAs. Tukey HSD post hoc test was used for RM ANOVAs, while for GLMs and GLMMs, post hoc multiple comparisons were performed with the R-package ‘multcomp’ [56]. For productivity and egg-to-adult viability (**S3 and S4 Figs**), where post hoc multiple comparisons were performed, the P-values were corrected for multiple testing (66 pairwise comparisons for productivity and 28 for egg-to-adult viability) using sequential Bonferroni correction. Nucleotide diversity was compared across inbreeding levels and extinction groups using Welch’s t-tests.

As a measure of evolutionary response, we used the regression coefficients (slope) of the linear models of the response across generations. For each trait, we assessed the explanatory power of population bottleneck history versus nucleotide diversity as predictors of the evolutionary response (slope). We did this by comparing the coefficients of determination (R^2^) of two contrasting linear models; slope as a function of expected F or slope as a function of π. Furthermore, we fitted a Davidson & MacKinnon encompassing model [57] where the two competing models (π or F as the predictor variable) used to predict the evolutionary response, are nested in a combined model. A Wald test comparing each of the individual models with the encompassing model was then carried out to evaluate the predictive performance of each variable. Encompassing models and Wald tests were performed using the R-package ‘lmtest’ [58].

## Supporting information

S1 Table Number of experimental lines per generation. Number of lines for each generation and for each level of inbreeding (Low; L, Medium; M, and High; H) and for the outbred control lines (OB). Bottom row shows total number of lines per generation.
(XLSX)

S2 Table Productivity for generations 1–10. Productivity (line means) for 122 lines of the three different inbreeding (F) levels (High; H, Medium; M, and Low; L) and for the outbred lines (OB) for generations 1–10. For productivity, we only have one measure per line per generation, so SD cannot be determined.
(XLSX)

S3 Table Dry body mass for generations 1–10. Dry body mass (line means) from 122 lines of the three different inbreeding (F) levels (High; H, Medium; M, and Low; L) and from the outbred lines (OB). Mean, n, and SD are shown for generations 1–10.
(XLSX)

S4 Table Egg-to-adult viability before and after the experiment. Results of baseline characterization of egg-to-adult viability (%) of 122 lines from the three different inbreeding (Inb.) levels (High; H, Medium; M, and Low; L) and from 10 outbred lines (OB1-OB10) from five treatments ranging from a benign standard medium (‘Control’) to four stressful media consisting of 9.5 g/L yeast, 16 g/L agar and 1.0, 2.5, 5.0 or 10.0 mL/L acetic acid, designated A1, A2, A3 and A4, respectively. The acetic acid concentration yielding the survival closest to 50% egg-to-adult viability for a given line was selected as the acid concentration used in the evolution experiment for that line, and is given in the ‘A(s)’ column. The baseline experiment was set up in five replicate vials as described in the text. The ‘Ext.’ column designates lines that went extinct during the experiment. Egg-to-adult viability (%) was assessed after the 10 generations on the stressful medium (on which the specific line had been reared), and at a benign medium. Results are shown in the last two columns (denoted ‘Benign’ and ‘Stress’) for the 96 lines that persisted through the experiment.
(XLSX)

S5 Table Summary of nucleotide diversity within inbreeding levels. Summary of nucleotide diversity (π) of the experimental lines of the three inbreeding levels (Low; L, Medium; M, and High; H), and of the outbred (OB) control lines. Mean, standard deviations (SD), minimum and maximum π values are expressed as relative to the mean of the OB control lines (index 1.0). The number of lines for which π could be obtained is shown (total n = 119).
(XLSX)

S6 Table Number of SNPs per line used to estimate nucleotide diversity. Number of SNPs (No. SNPs evaluated) used to estimate nucleotide diversity (π), of the experimental lines of the three inbreeding levels (Low; L, Medium; M, and High; H) and of the outbred (OB) control lines. Only lines for which π could be obtained is shown (total n = 119).
(XLSX)

S1 Fig Flow-chart of experimental procedure. Experimental procedure from setting up inbreeding regimes, to measuring initial stress responses and collecting samples for nucleotide diversity, followed by 10 generations of exposure to stressful medium, and lastly assessment of egg-to-adult viability. The stressful medium was line-specific, i.e. the acetic acid concentration yielding the survival closest to 50% egg-to-adult viability in the initial baseline characterization of the stress response was selected as the acid concentration used in the experimental evolution study. *Egg-to-adult viability was assessed after 10 generations on the stressful medium on which the specific line had been reared, and on a benign medium. These viability measures were compared to the egg-to-adult viability from the baseline response, to identify adaptive responses for this trait. Productivity and dry body mass were assessed every generation. See text for details on each step in the procedure.
(PNG)

S2 Fig Evolutionary response for all lines for body mass and productivity. Line plots of all lines across generations 1–10 within each inbreeding group: High (**A-B**; in red), Medium (**C-D**; in orange), Low (**E-F**; in yellow), and outbred lines (**G-H**; in blue), and all lines plotted together (**I-J**) for body mass (left side), and productivity (right side). Y-axes are similar for all plots within a trait for ease of comparison.
(PNG)

S3 Fig Evolutionary response within inbreeding levels for body mass and productivity. Response in (**A**) body mass, and (**B**) productivity across generations 1–10 for the three inbreeding levels (Low; L (yellow), Medium; M (orange), and High; H (red)), and the outbred lines (OB (blue)). Error bars represent SE. The number of lines at each generation can be seen in S1 Table. Different letters denote significantly different groups at selected generations 1, 6, and 10, as based on post hoc multiple comparisons test (P < 0.05; P-values were corrected for multiple testing with Tukey HSD post hoc tests for body mass and Bonferroni correction for productivity (66 pairwise comparisons)).
(PDF)

S4 Fig Differences in egg-to-adult viability before and after experimental evolution. Differences in egg-to-adult viability (in %) before and after 10 generations of experimental evolution for the three inbreeding levels (Low; L (yellow), Medium; M (orange), and High; H (red)), and the outbred lines (OB (blue)). Values are expressed as after the experiment (F10) compared to before (F0), i.e. a negative value means that the viability is lower after ending the experiment. Values are expressed as the mean of the difference for each line, rather than the difference in means across all lines, to correctly reflect the between-line variation. Error bars represent the SE of this difference, which is calculated from the variance sum law as described in the methods section. Asterisks denote differences that are not significantly different from 0 (P < 0.05). Letters denote significant differences across inbreeding levels and across types of medium. All P-values were corrected for multiple testing using Bonferroni correction (28 pairwise comparisons).
(PNG)

S5 Fig Correlations between nucleotide diversity and evolutionary responses within inbreeding levels. Correlations between nucleotide diversity (π) and slopes (a measure of evolutionary responses) for productivity (**A**) and dry body mass (**B**) for the three inbreeding levels (Low; L (yellow; diamonds), Medium; M (orange; circles), and High; H (red; triangles)), and the outbred lines (OB (blue; squares)). For all regressions, R^2^ values are shown, followed by asterisks denoting significant correlations. For body mass, Pearson’s product-moment correlations were used, and for productivity, Spearman’s rank correlations were used. The solid lines represent the linear regressions to visualize the correlation. We only considered slopes for lines that did not go extinct. This was done to ensure that unreliable slopes (estimated based on information from e.g. just 2 generations) were not included; in total this yielded slope estimates for 87 lines.
(PNG)

S6 Fig Coefficient of variation across generations for body mass and productivity. Coefficients of variation (CV in %) across lines for (**A**) body mass, and (**B**) productivity across generations 1–10 for the three inbreeding levels (Low; L (yellow), Medium; M (orange), and High; H (red)), and the outbred lines (OB (blue)).
(PNG)

S7 Fig Cumulative distributions of read coverage for each population bottleneck treatment. Cumulative distributions of read coverage for each population bottleneck treatment (CumLow, CumMedium, and CumHigh) and of the outbred (CumOB) control group. To test if the read coverage distributions were similar, a Kolmogorov-Smirnov test was performed. At α = 0.05, the distributions were not significantly different between population bottleneck treatments.
(PNG)
