# Supplementary figures and images for: Genomic variation predicts adaptive evolutionary responses better than population bottleneck history
Source: PLoS Genet. 2019 Jun 12;15(6):e1008205. doi: 10.1371/journal.pgen.1008205 (PMC6590832; doi:10.1371/journal.pgen.1008205)

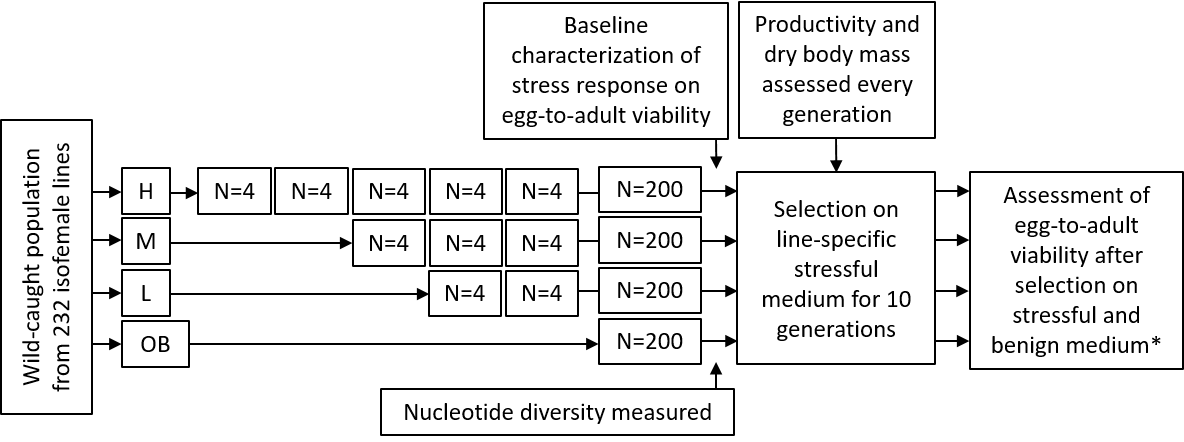

Supplement: S1 Fig — Experimental procedure from setting up inbreeding regimes, to measuring initial stress responses and collecting samples for nucleotide diversity, followed by 10 generations of exposure to stressful medium, and lastly assessment of egg-to-adult viability. The stressful medium was line-specific, i.e. the acetic acid concentration yielding the survival closest to 50% egg-to-adult viability in the initial baseline characterization of the stress response was selected as the acid concentration used in the experimental evolution study. *Egg-to-adult viability was assessed after 10 generations on the stressful medium on which the specific line had been reared, and on a benign medium. These viability measures were compared to the egg-to-adult viability from the baseline response, to identify adaptive responses for this trait. Productivity and dry body mass were assessed every generation. See text for details on each step in the procedure. (PNG) [file pgen.1008205.s007.png]

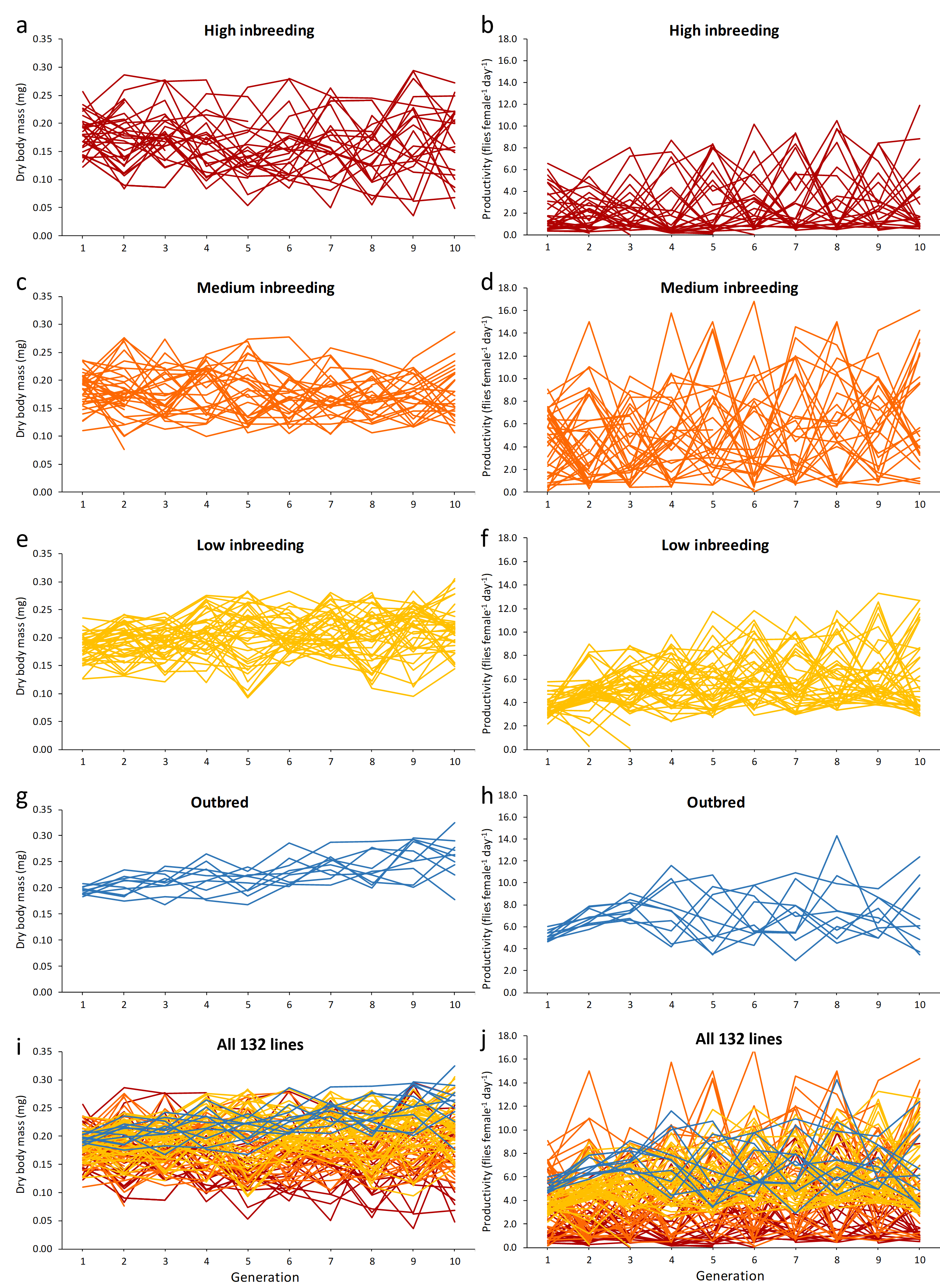

Supplement: S2 Fig — Line plots of all lines across generations 1–10 within each inbreeding group: High (A-B; in red), Medium (C-D; in orange), Low (E-F; in yellow), and outbred lines (G-H; in blue), and all lines plotted together (I-J) for body mass (left side), and productivity (right side). Y-axes are similar for all plots within a trait for ease of comparison. (PNG) [file pgen.1008205.s008.png]

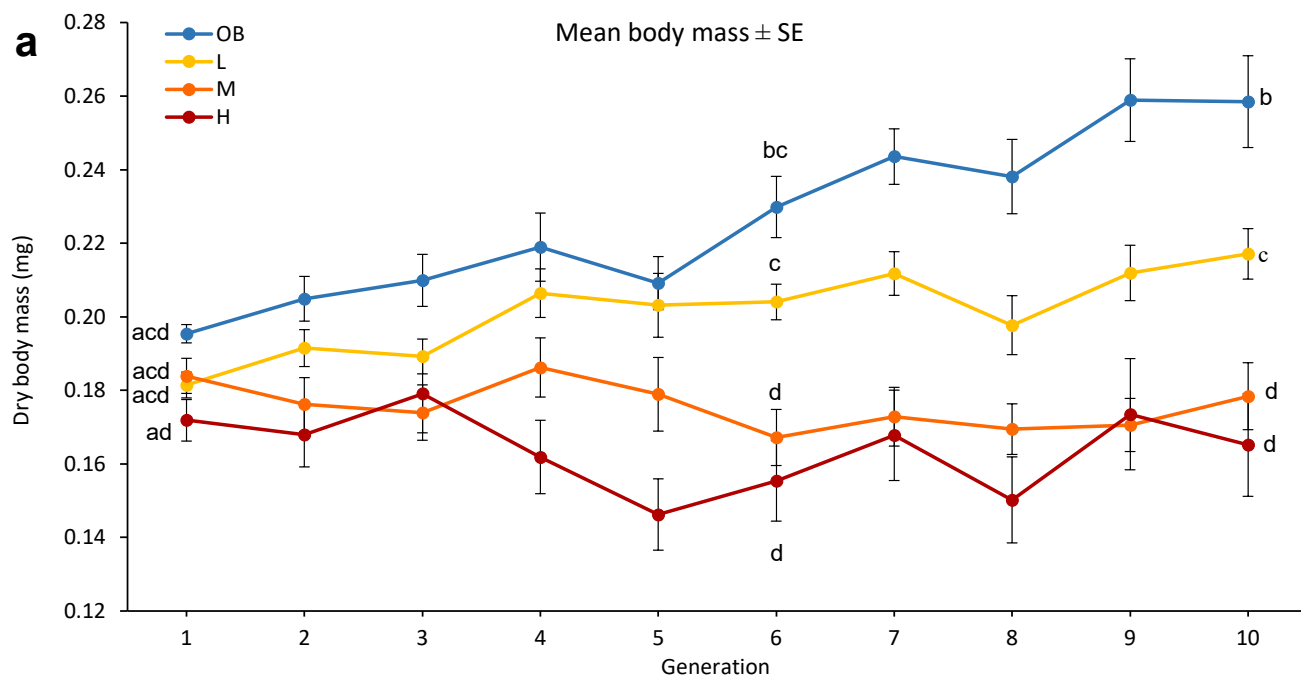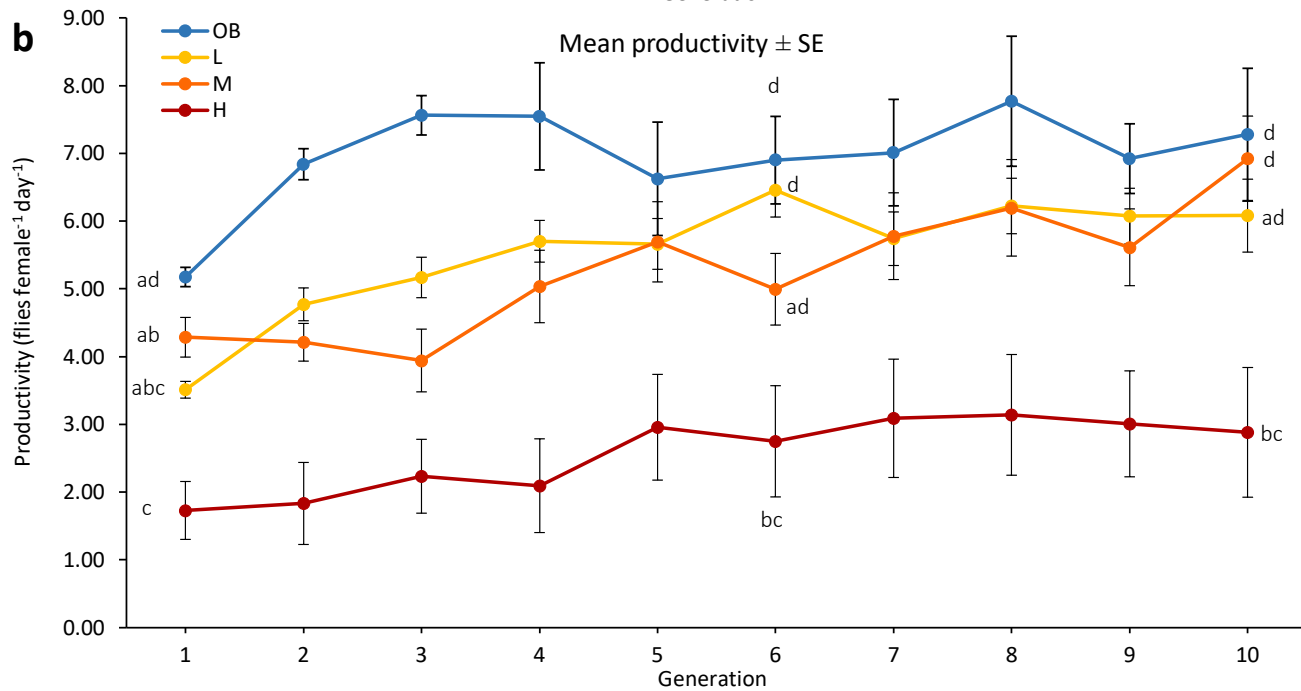

Supplement: S3 Fig — Response in (A) body mass, and (B) productivity across generations 1–10 for the three inbreeding levels (Low; L (yellow), Medium; M (orange), and High; H (red)), and the outbred lines (OB (blue)). Error bars represent SE. The number of lines at each generation can be seen in S1 Table. Different letters denote significantly different groups at selected generations 1, 6, and 10, as based on post hoc multiple comparisons test (P < 0.05; P-values were corrected for multiple testing with Tukey HSD post hoc tests for body mass and Bonferroni correction for productivity (66 pairwise comparisons)). (PDF) [file pgen.1008205.s009.pdf]

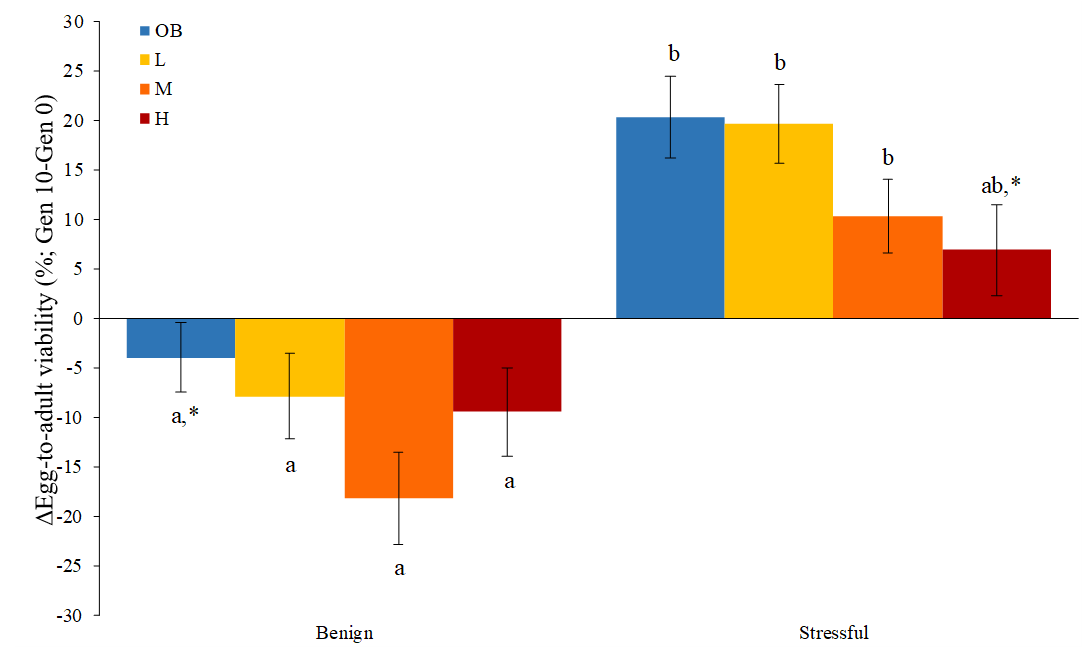

Supplement: S4 Fig — Differences in egg-to-adult viability (in %) before and after 10 generations of experimental evolution for the three inbreeding levels (Low; L (yellow), Medium; M (orange), and High; H (red)), and the outbred lines (OB (blue)). Values are expressed as after the experiment (F10) compared to before (F0), i.e. a negative value means that the viability is lower after ending the experiment. Values are expressed as the mean of the difference for each line, rather than the difference in means across all lines, to correctly reflect the between-line variation. Error bars represent the SE of this difference, which is calculated from the variance sum law as described in the methods section. Asterisks denote differences that are not significantly different from 0 (P < 0.05). Letters denote significant differences across inbreeding levels and across types of medium. All P-values were corrected for multiple testing using Bonferroni correction (28 pairwise comparisons). (PNG) [file pgen.1008205.s010.png]

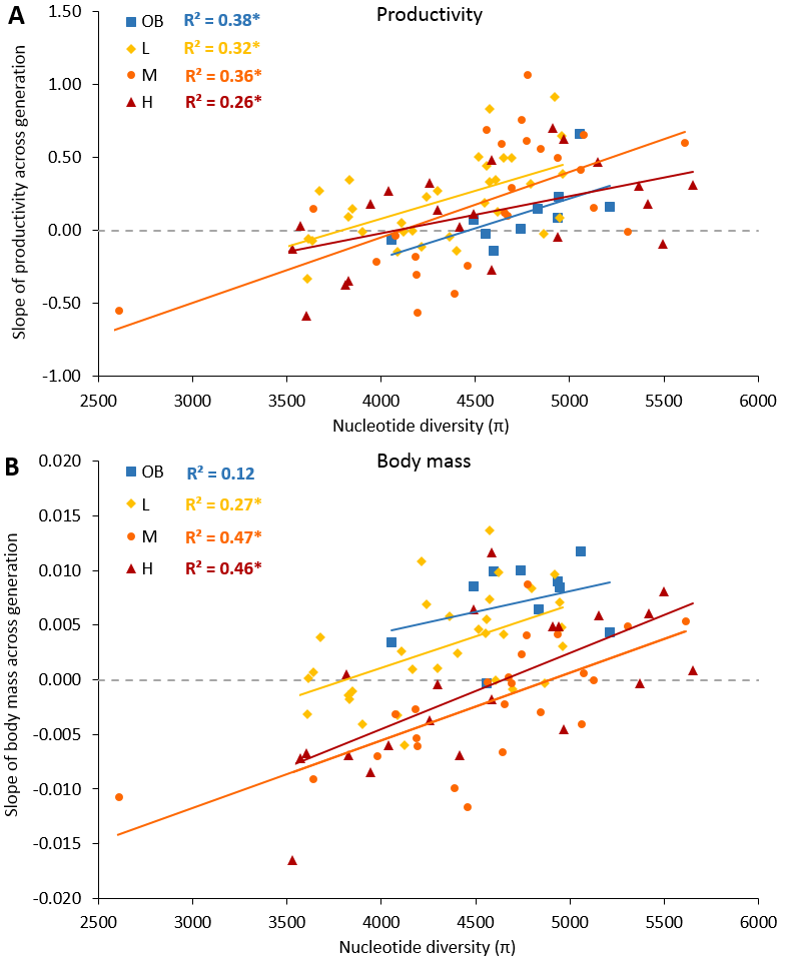

Supplement: S5 Fig — Correlations between nucleotide diversity (π) and slopes (a measure of evolutionary responses) for productivity (A) and dry body mass (B) for the three inbreeding levels (Low; L (yellow; diamonds), Medium; M (orange; circles), and High; H (red; triangles)), and the outbred lines (OB (blue; squares)). For all regressions, R2 values are shown, followed by asterisks denoting significant correlations. For body mass, Pearson’s product-moment correlations were used, and for productivity, Spearman’s rank correlations were used. The solid lines represent the linear regressions to visualize the correlation. We only considered slopes for lines that did not go extinct. This was done to ensure that unreliable slopes (estimated based on information from e.g. just 2 generations) were not included; in total this yielded slope estimates for 87 lines. (PNG) [file pgen.1008205.s011.png]

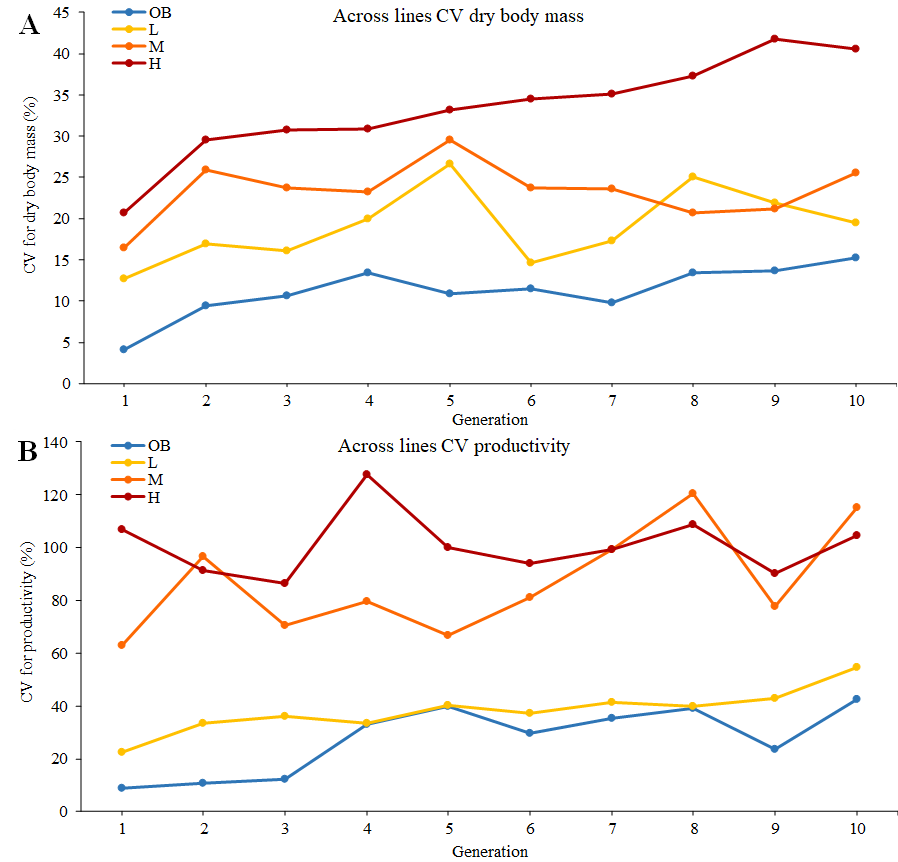

Supplement: S6 Fig — Coefficients of variation (CV in %) across lines for (A) body mass, and (B) productivity across generations 1–10 for the three inbreeding levels (Low; L (yellow), Medium; M (orange), and High; H (red)), and the outbred lines (OB (blue)). (PNG) [file pgen.1008205.s012.png]

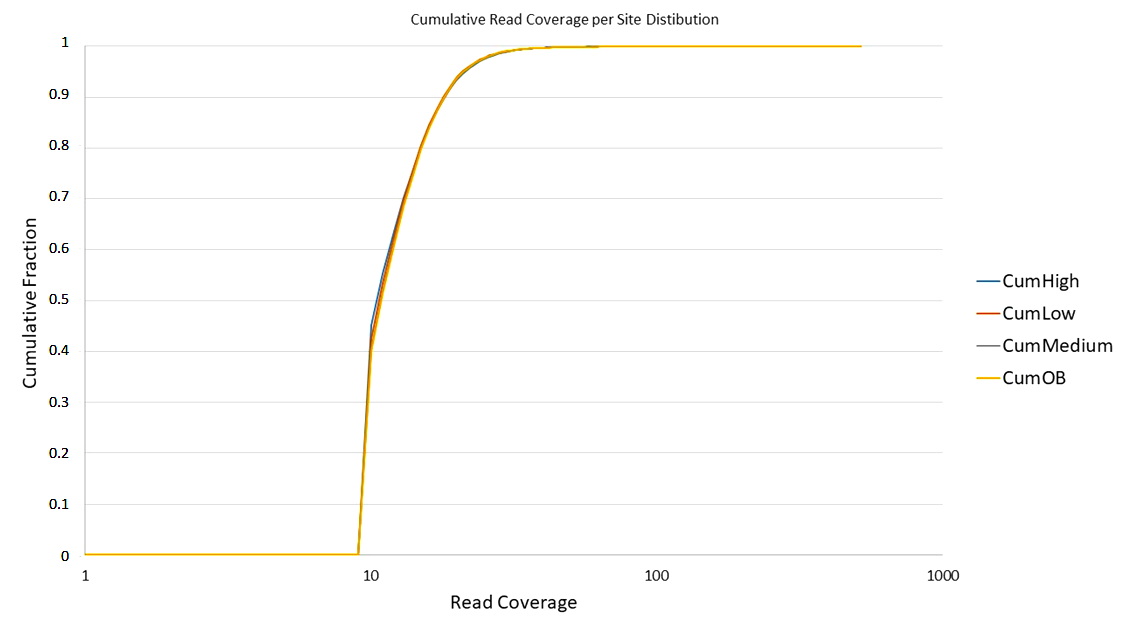

Supplement: S7 Fig — Cumulative distributions of read coverage for each population bottleneck treatment (CumLow, CumMedium, and CumHigh) and of the outbred (CumOB) control group. To test if the read coverage distributions were similar, a Kolmogorov-Smirnov test was performed. At α = 0.05, the distributions were not significantly different between population bottleneck treatments. (PNG) [file pgen.1008205.s013.png]
